# Promotion of Bone Lesions Through the Myeloma Integrin α6-Mediated Osteolytic Signaling

**DOI:** 10.3389/fonc.2021.692190

**Published:** 2021-06-03

**Authors:** Huan Liu, Zhiming Wang, Jin He, Zongwei Li, Jerry Y. Gao, Rui Liu, Pei Lin, Jing Yang

**Affiliations:** ^1^ Houston Methodist Cancer Center, Houston Methodist Research Institute, Houston Methodist Hospital, Houston, TX, United States; ^2^ Cancer Research Center, School of Medicine, Xiamen University, Xiamen, China; ^3^ Department of Hematopathology, Division of Pathology and Laboratory Medicine, The University of Texas MD Anderson Cancer Center, Houston, TX, United States

**Keywords:** multiple myeloma, bone lesion, integrin α6, laminin, EGFR

## Abstract

Osteolytic destruction is a hallmark of multiple myeloma and impairs myeloma patients’ quality of life. However, the molecular mechanism underlying the pathogenesis of myeloma-associated bone disease remains unclear. In this study, we demonstrate the role of myeloma cell-expressed integrin α6 in bone. Integrin α6 binds to laminin 8 and epidermal growth factor receptor on mesenchymal stem cells (MSCs) to form a trimer complex and upregulates the secretion of osteolytic cytokines from both myeloma cells and MSCs, leading to enhanced bone resorption and reduced bone formation. Thus, this study elucidates an important mechanism for myeloma-induced bone lesions and implicates that targeting integrin α6 may be a viable approach for bone healing in myeloma patients.

## Introduction

Multiple myeloma is the second most common hematologic malignancy in the United States. It is a plasma cell malignancy characterized by the clonal expansion of malignant plasma cells within bone marrow (1). One hallmark of multiple myeloma is osteolytic lesions (2). More than eighty percentage of patients with myeloma suffer from skeleton-related events, which includes pathological fracture, severe bone pain, spinal cord compression, and hypercalcemia, severely impacting their quality of life (1). The standard treatment for myeloma patients with bone disease relies on drugs like bisphosphonates to alleviate bone loss. However, those therapies only offer moderate palliative effects. Therefore, an urgent need for the discovery of new therapeutic target is warranted.

Myeloma-induced bone destruction stems from disrupted balance in bone remodeling, and the resorbed bone in myeloma patients rarely heals. In patients with myeloma-associated bone disease, increased bone resorption is mediated by osteoclasts that arise from hematopoietic monocytic precursors (2, 3). New bone formation in patients is also suppressed by myeloma cells through inhibition of osteoblast differentiation from mesenchymal stem cells (MSCs) (2, 3). Myeloma cells can secrete osteolytic cytokines or stimulate their release by surrounding MSCs. For example, receptor activator of nuclear factor-κB ligand (RANKL) can enhance osteoclast differentiation, while dickkopf-related protein 1 (DKK1) inhibits the Wnt/β-catenin signaling pathway and suppresses osteoblast differentiation (3). But attempts to target RANKL and DKK1 in myeloma therapeutically have only achieved modest success (2), suggesting that there are other key mechanisms awaiting discovery.

Integrins are among the major families of cell adhesion receptors, and they are found in many animal species ranging from sponges to mammals. There are many types of integrins with each composing of α and β subunits, and the unique combination of subunits determines its specific binding and signaling properties. Integrins relay signals that are essential for intercellular communication, with signals in-and-out of the plasma membrane between the extracellular matrix and cell-surface ligands as well as cytoskeletal and signaling effectors (4, 5). They have also been shown to be involved in many aspects of cancer development. Their promotion of tumor cell migration, invasion, proliferation, and survival contributes to the increased tumor progression and metastasis (6). For instance, integrin subunit α6, also named as ITGA6, plays a role in melanoma cell metastasis and glioblastoma stem cell self-renewal (7, 8). In head and neck carcinoma, the expression of integrin α6 is correlated with poor prognosis in patients (9). Structurally, integrin α6 can form heterodimers with either β1 or β4 subunit to α6β1 and α6β4 integrins, respectively (10). However, the role of integrin α6 in the pathogenesis of myeloma bone disease remains unclear.

In this study, we investigate the interaction between MSCs and myeloma cells mediated by integrin α6. Our results show that myeloma cell integrin α6 binds to laminin 8 and epidermal growth factor receptor (EGFR) on MSCs, forms a trimer complex, activates the ERK1/2, STAT1/3, PI3K/Akt signaling pathways, enhances the secretion of osteolytic cytokines from myeloma cells and MSCs. Our findings not only elucidate a mechanism of myeloma-induced suppression of osteoblast differentiation and activation of osteoclast differentiation, but also implicate a potential therapeutic approach for bone healing in myeloma patients by targeting integrin α6.

## Materials and Methods

### Cell Lines and Primary Myeloma Cells

The myeloma cell line ARP-1 was provided by the University of Arkansas for Medical Sciences (Little Rock, Arkansas, USA). HEK293T and RPMI8226 cells were purchased from the American Type Culture Collection (Manassas, VA, USA). Primary myeloma cells were isolated from bone marrow aspirates from patients with myeloma using anti-CD138 antibody-coated magnetic beads (Miltenyi Biotec, Bergisch Gladbach, Germany). Myeloma cells were maintained in RPMI 1640 medium with 10% fetal bovine serum (FBS), and HEK293T cells were cultured in Dulbecco’s modified Eagle’s medium (DMEM) with 10% FBS. Patient samples were obtained from UT MD Anderson Cancer Center and Biorepository of Houston Methodist Research Institute. Bone lesions in the study participants were characterized by radiologists from Bone Survey at UT MD Anderson Cancer Center. The radiologists were blinded to the severity of clinical disease. This study was approved by the Institutional Review Boards of UT MD Anderson Cancer Center and Houston Methodist Research Institute.

### Antibodies, Plasmids, and Reagents

The plasmids *EGFR-GFP* and *ITGA6-His* were purchased from GeneCopoeia (Rockville, Maryland, USA). Except where specified, all chemicals were purchased from Sigma-Aldrich (St. Louis, Missouri, USA), and all antibodies for Western blot analysis were purchased from Cell Signaling Technology (Danvers, Massachusetts, USA). ELISA kits were purchased from R&D Systems (Minneapolis, Minnesota, USA). shRNAs against integrin α6 and non-target control were purchased from Sigma-Aldrich.

### *In Vitro* Osteoblast and Osteoclast Formation and Function Assays

MSCs were obtained from the bone marrow of healthy donors, and mature osteoblasts were generated from MSCs with osteoblast medium as described previously (3, 11). The bone formation activity of osteoblasts was determined using Alizarin red S (Sigma-Aldrich) staining as described previously (3, 11). Human monocytes were isolated from peripheral blood mononuclear cells of health donors and cultured as described previously to obtain the precursors of osteoclasts (3, 11). The precursors derived from human monocytes were cultured in M-CSF (25 ng/ml), a low dose of RANKL (5 ng/ml) or myeloma cells for 7 days to induce mature osteoclast formation. TRAP staining for the detection of mature osteoclasts was performed using a leukocyte acid phosphatase kit (Sigma-Aldrich) according to the manufacturer’s instructions.

### Western Blot Analysis

Cells were harvested and lysed with 1 × lysis buffer (Cell Signaling Technology). Cell lysates were subjected to SDS-PAGE, transferred to a polyvinylidene difluoride (PVDF) membrane, and immunoblotted with antibodies against LAMA4 (Santa Cruz Biotechnology, Cat # sc-130541, RRID : AB_2296778), GAPDH (Thermo Fisher Scientific, Cat # AM4300, RRID: AB_2536381), EGFR (Cell Signaling Technology Cat# 4267, RRID : AB_2246311), ITGA6 (Santa Cruz Biotechnology Cat# sc-374057, RRID : AB_10917002), β-actin (Santa Cruz Biotechnology, Cat # sc-69879, RRID: AB_1119529), phosphorylated ERK1/2 (Cell Signaling Technology Cat# 9101, RRID : AB_331646), STAT1 (Cell Signaling Technology Cat# 9167, RRID : AB_561284), STAT3 (Cell Signaling Technology Cat# 9145, RRID : AB_2491009), and Akt (Cell Signaling Technology Cat# 9271, RRID : AB_329825), non-phosphorylated ERK1/2 (Cell Signaling Technology Cat# 4695, RRID : AB_390779), STAT1 (Cell Signaling Technology Cat# 14994, RRID : AB_2737027), STAT3 (Cell Signaling Technology Cat# 9132, RRID : AB_331588) and Akt (Cell Signaling Technology Cat# 9272, RRID : AB_329827).

### Quantitative Real-Time PCR

Total RNA was isolated using a RNeasy kit (QIAGEN). An aliquot of 1 µg of total RNA was subjected to reverse transcription (RT) with a SuperScript II RT-PCR kit (Life Technologies, Carlsbad, California, USA) according to the manufacturer’s instructions. Quantitative PCR was performed using SYBR Green Master Mix (Life Technologies) with the QuantStudio 3 Real-Time PCR System (Life Technologies).

### Immunoprecipitation Assay

Cells were lysed and incubated on ice for 15 min. The total protein lysate was immunoprecipitated with an agarose-immobilized antibody at 4°C overnight. After washing six times, the beads were spun down and resuspended in SDS loading buffer. Pull-down samples were run on a SDS-PAGE gel along with a 5% input sample and transferred to a PVDF membrane for immunoblotting. IgG was used as a control and total cell lysates were used as input controls.

### *In Vivo* Mouse Experiments, Measurement of Tumor Burden, Radiography, and Bone Histomorphometry

CB.17 SCID mice purchased from Envigo (Indianapolis, Indiana, USA) were maintained in American Association of Laboratory Animal Care–accredited facilities. The mouse studies were approved by the Institutional Animal Care and Use Committee of UT MD Anderson Cancer Center and The Houston Methodist Hospital. Cultured myeloma cells (5×10^5^ cell/mouse) were injected into the femurs of 6- to 8-week-old SCID mice. To monitor the tumor burden, serum samples were collected from the mice weekly and tested for the presence of myeloma-secreted M proteins using ELISA analysis. Bone tissues were fixed in 10% neutral-buffered formalin and decalcified, and sections of them were stained with toluidine blue or TRAP following standard protocols. Both analyses were done using the BIOQUANT OSTEO (v18.2.6) software program (BIOQUANT Image Analysis Corporation, Nashville, Tennessee, USA).

### Statistical Analysis

Statistical significance was analyzed using the SPSS software program (version 10.0; IBM Corporation) with two tailed unpaired Student *t*-tests for comparison of two groups, and one-way ANOVA with Tukey’s multiple comparisons test for comparison of more than two groups. *P* values less than 0.05 were considered statistically significant. All results were reproduced in at least three independent experiments.

## Results

### Highly Expressed Integrin α6 in Myeloma Cells Is Positively Associated With Bone Lesions in Patients

**Figure 1:**
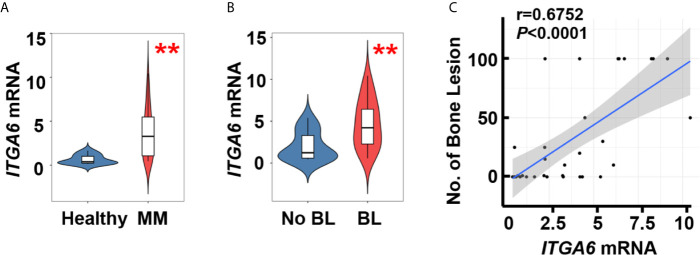
Integrin α6 is positively associated with lytic lesions in myeloma patients. **(A)** Quantitative PCR analysis of *ITGA6* mRNA levels in normal plasma cells from healthy donors (n = 10) and malignant plasma cells from myeloma patients (MM, n = 34). **(B)** *ITGA6* mRNA levels in malignant plasma cells of 13 myeloma patients without bone lesion (BL = 0) and 21 myeloma patients with bone lesion (BL ≥ 1). Data shown as averages ± SD. **P < 0.01.* P* values were determined by Student’s *t* test **(A, B)**. **(C)** Correlation coefficient of the mRNA levels of *ITGA6* and numbers of bone lesion in myeloma patients (n = 34). The correlations were evaluated using Pearson coefficient. r, correlation coefficient. *P* value was determined by Pearson coefficient.

We first examined the expression of integrin α6 in patients with newly diagnosed multiple myeloma, quantitative polymerase chain reaction (qPCR) analysis showed significant upregulation in malignant plasma cells isolated from bone marrow aspirates of those patients (n=34) when compared with those in plasma cells from normal subjects (n=10, **Figure 1A**). We then divided myeloma patients into two groups on whether they suffered from bone lesions. The levels of integrin α6 expression were higher in myeloma cells of patients with bone lesions compared with those without (**Figure 1B**). Furthermore, we found a strong positive correlation between the level of integrin α6 expression in myeloma cells and bone lesion numbers in patients (**Figure 1C**). These results indicate the association of integrin α6 to myeloma bone disease.

### Myeloma Cell Integrin α6 Enhances Bone Lesions Through Inhibition of Osteoblastogenesis and Activation of Osteoclastogenesis

**Figure 2:**
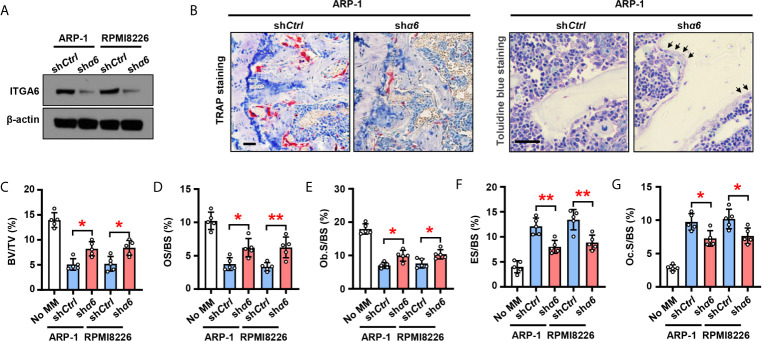
Myeloma cells integrin α6 inhibits osteoblast differentiation and enhances osteoclast differentiation *in vivo*. Myeloma cell lines ARP-1 or RPMI 8226 were infected with lentivirus carrying nontargeted shRNA (sh*Ctrl*) or *ITGA6* shRNA (sh*α6*). **(A)** Western blotting shows the level of ITGA6 in sh*Ctrl* or sh*α6* myeloma cells. Levels of β-actin served as loading control. Representative images are shown from three independent experiments. **(B–G)** SCID mice were injected sh*Ctrl* or sh*α6* myeloma cells. Mice without myeloma cell injection served as baseline parameters for bone analysis. After 4 weeks, mouse femurs were extracted, fixed, TRAP- or toluidine blue-stained, and analyzed. **(B)** Representative images of TRAP- or toluidine blue-stained femurs of myeloma-bearing mice. Scale bar: 50 μm. **(C–G)** Shown are the percentage of bone volumes versus total volumes (BV/TV) **(C)**, the percentages of osteoid surface (OS/BS) **(D)** and total bone surface lined with osteoblasts (Ob.S/BS) **(E)**, the percentages of bone surface eroded by osteoclasts (ES/BS) **(F)** and bone surface covered with osteoclasts (Oc.S/BS) **(G)**. Data are means ± SD (*n* = 5 mice/group, three replicate studies). *P < 0.05; **P < 0.01. *P* values were determined using one-way ANOVA.

To examine the functional role of integrin α6 in bone, we knocked down its expression in ARP-1 or RPMI8226 myeloma cells using small hairpin RNAs (shRNAs) against human α6 (**Figure 2A**) and then injected myeloma cells into the femurs of NSG mouse. Bone histomorphometric analysis (**Figure 2B**) showed higher percentages of bone volume/total volume (BV/TV) (**Figure 2C**), osteoid surface (OS/BS) (**Figure 2D**), bone surface lined with osteoblasts (Ob.S/BS) (**Figure 2E**), and lower percentages of bone surface eroded by osteoclasts (ES/BS) (**Figure 2F**), bone surface covered with osteoclasts (Oc.S/BS) (**Figure 2G**) in the mice injected with sh*α6* myeloma cells than those in mice with non-target control (sh*Ctrl*) cells. These results indicate that integrin α6 contributes to myeloma-induced bone lesions.

**Figure 3:**
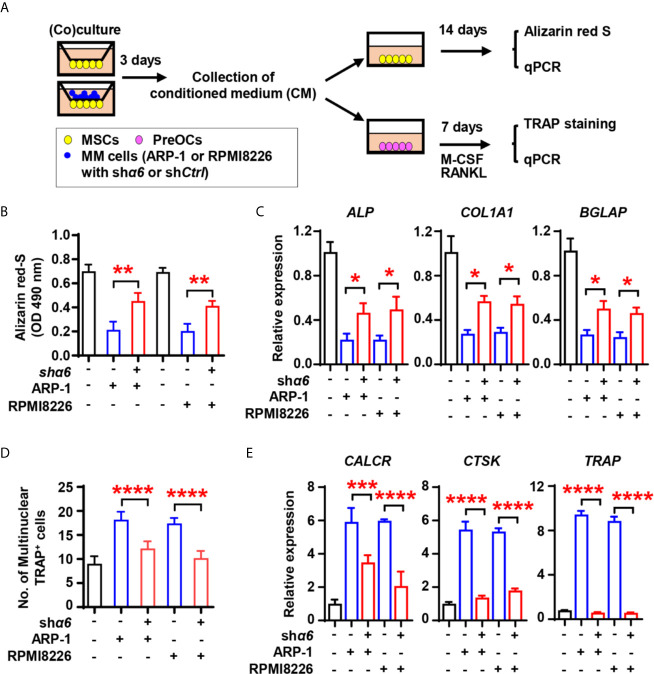
Myeloma cell integrin α6 inhibits osteoblast differentiation *in vitro*. **(A)** The schematic of coculture systems of MSCs with myeloma cells. **(B, C)** MSCs were cocultured with myeloma cells. RNAs were extracted for qPCR analysis of osteoblast marker genes and cells were stained with Alizarin red S staining to assess osteoblastogenesis. Shown are the summarized data of Alizarin red S staining **(B)**, and the relative expression of *BGLAP*, *ALP*, and *COL1A1* genes in MSCs **(C)**. **(D, E)** preOCs were cocultured with myeloma cells. RNAs were extracted for qPCR analysis and cells were stained with TRAP to access osteoclastogenesis. Shown are the schematic of coculture systems of preOCs with myeloma cells **(D)**, numbers of multinuclear (≥3) TRAP^+^ cells **(E)**, and the relative expression of *CALCR*, *CTSK*, and *TRAP* in precursors of osteoclasts. Data shown as averages ± SD is a representative of three independent experiments. *P < 0.05. **P < 0.01. ***P < 0.001. ****P < 0.0001. All *P* values were determined using one-way ANOVA.

We next assessed the effect of myeloma cell integrin α6 on osteoblast formation *in vitro*. As shown schematically in **Figure 3A**, we cultured MSCs alone or coculture MSCs with sh*Ctrl*- or sh*α6*-expressing myeloma cells for 3 days and collected the conditioned medium (CM). We then cultured a fresh MSCs as the osteoblast progenitors in osteoblast medium with or without addition of the CM. While adding the CM of MSCs cocultured with myeloma cells reduced Alizarin red S staining when compared to those of MSCs alone (**Figure 3B**), we observed that the presence of CM of MSCs cocultured with sh*α6* myeloma cells caused more Alizarin red S staining than those in the CM of MSCs with sh*Ctrl* myeloma cells (**Figure 3B**). The expression of osteoblast differentiation-associated genes was also enhanced in the presence of CM with sh*α6* myeloma cells (**Figure 3C**). To study the ability of myeloma integrin α6 in regulating osteoclast differentiation *in vitro*, we cultured monocyte-derived preosteoclasts (preOCs) with the CM for 7 days (**Figure 3A**). Following standard protocol, we stained the cells with tartrate-resistant acid phosphatase (TRAP), and we confirmed that adding the CM of MSCs cocultured with myeloma cells enhanced TRAP^+^ cell numbers and osteoclast-associated gene expression comparing to those with addition of MSC CM, while knockdown of integrin α6 in myeloma cells reduced such effects (**Figures 3D, E**). These results indicate that myeloma cell integrin α6 has effects on suppression of obsteoblastogenesis and activation of obsteoclastogenesis.

### Interaction Between Myeloma Cells and MSCs Upregulates Osteolytic Cytokine Production Through Integrin α6-Mediated Signaling Pathways

**Figure 4:**
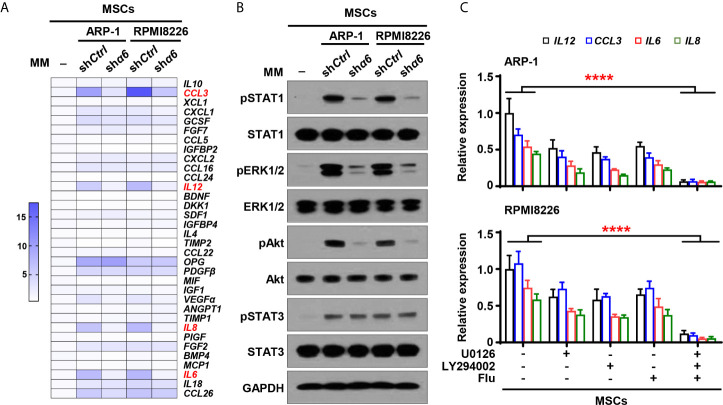
Myeloma cell integrin α6 regulates cytokine expression *via* Akt, ERK, and STAT1 signaling pathways in MSCs. MSCs were cocultured with or without myeloma cell lines ARP-1 or RPMI 8226 carrying nontargeted shRNA (*shCtrl*) or *ITGA6* shRNA (*shα6*) for 24 hours. **(A)** Quantitative real-time PCR analysis shows the relative expression of cytokine genes in MSCs. **(B)** Western blotting shows phosphorylated (p), or non-phosphorylated ERK1/2, Akt, STAT1 and STAT3 in MSCs. GAPDH levels served as protein loading controls. Representative images are shown from three independent experiments. **(C)** The relative expression of *IL12*, *CCL3*, *IL6* and *IL8* mRNAs in MSCs co-cultured with ARP-1 (Upper panel) or RPMI8226 (Lower panel) in the absence or presence of the STAT1 inhibitor Fludarabine (10 µM), ERK inhibitor U0126 (5 µM) or Akt inhibitor LY294002 (10 µM). Data shown as averages ± SD is a representative of three independent experiments. ****P < 0.0001. All *P* values were determined using one-way ANOVA.

To examine the functional role of myeloma cell integrin α6 in osteolytic cytokines expression in MSCs, we cocultured MSCs with sh*α6* or sh*Ctrl* myeloma cells for 2 days and screened a panel of 34 cytokines or chemokines that involve in osteoblast and osteoclast differentiation. Using qPCR analysis, we identified top four cytokines: inflammatory protein 1-alpha (MIP-1α), interleukin (IL)-6, -8, and -12, which were not only upregulated in MSCs cocultured with myeloma cells compared to MSCs alone, but was significantly downregulated in MSCs exposed to integrin α6 knocked down myeloma cells when compared with that in MSCs exposed to control myeloma cells (**Figure 4A**).

We then examined several signaling pathways in MSCs, and we observed increased phosphorylation of extracellular signal–regulated kinase 1/2 (ERK1/2), signal transducer and activator of transcription (STAT) 1, STAT3, and Akt in MSCs cocultured with myeloma cells than those in MSCs alone (**Figure 4B**). Knockdown of integrin α6 in myeloma cells nearly diminished the activation effect from myeloma cells on the phosphorylation of ERK1/2, STAT1, and Akt in MSCs, except STAT3 (**Figure 4B**). To determine the impact of those signaling pathways on MSC cytokine expression, we added the inhibitor targeting ERK1/2, STAT1, or Akt to cocultures of MSCs and myeloma cells. Incubation with the kinase inhibitor, singly or in combination, reduced the expression of MIP-1α, IL-12, IL-8, and IL-6 in MSCs cocultured with myeloma cells (**Figure 4C**). Our findings indicate that myeloma cells upregulate cytokine expression in MSCs *via* the integrin α6-activated ERK1/2, Akt, and STAT1 signaling pathways.

**Figure 5:**
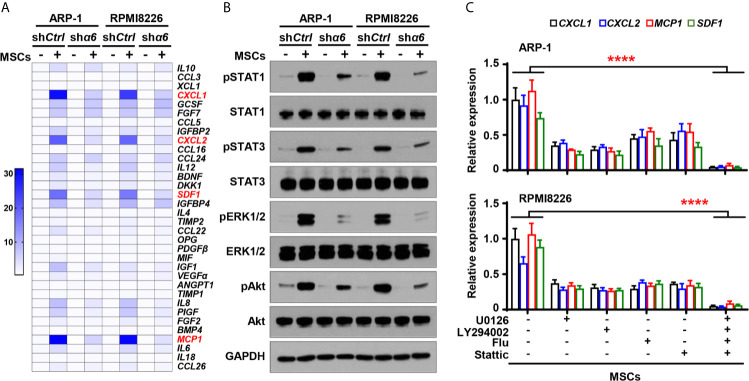
Integrin α6 regulates myeloma cell cytokines expression *via* Akt, ERK, STAT1 and STAT3 signaling pathways activated by MSCs. Myeloma cells ARP-1 or RPMI8226 carrying nontargeted shRNA (*shCtrl*) or*ITGA6* shRNA (*shα6*) were cocultured with MSCs for 24 hours. **(A)** Quantitative real-time PCR analysis shows the relative expression of cytokine genes in ARP-1 or RPMI8226 cells. **(B)** Western blotting shows phosphorylated (p), or non-phosphorylated ERK1/2, Akt, STAT1 and STAT3 in ARP-1 or RPMI8226 cells. GAPDH levels served as protein loading controls. Representative images are shown from three independent experiments. **(C)** The relative expression of *CXCL1*, *CXCL2*, *MCP1* and *SDF1* mRNAs in ARP-1 cells (Upper panel) or RPMI8226 cells (Lower panel) cultured with MSCs in the absence or presence of the STAT1 inhibitor Fludarabine (10 µM), STAT3 inhibitor Stattic (5 µM), ERK inhibitor U0126 (5 µM) or Akt inhibitor LY294002 (10 µM). Data shown as averages ± SD is a representative of three independent experiments. ****P < 0.0001. All *P* values were determined using one-way ANOVA.

?>To determine if the cytokine expression in myeloma cells is affected by the interaction between integrin α6 and MSCs, we cocultured sh*α6* or sh*Ctrl* myeloma cells with MSCs for two days and examined the expression of the same panel of 34 cytokines in myeloma cells using qPCR analysis. Of the top highly upregulated genes, we observed that the expression of four chemokines or cytokines, macrophage C-X-C motif ligand (CXCL)1, CXCL2, stromal cell-derived factor 1 (SDF1), and monocyte chemoattractant protein 1 (MCP1), were upregulated in myeloma cells cocultured with MSCs. Knockdown of integrin α6 expression in myeloma cells significantly downregulated those cytokine expressions in myeloma cells (**Figure 5A**). In addition, we observed the increased phosphorylation of ERK1/2, STAT1, STAT3, and Akt in myeloma cells cocultured with MSCs, while knockdown of integrin α6 in myeloma cells reduced those kinase phosphorylation (**Figure 5B**), suggesting that several signaling pathways were activated by integrin α6 in myeloma cells. When we added the inhibitor specific for ERK1/2, STAT1, STAT3, or Akt to cocultures of myeloma cells and MSCs, we found that the kinase inhibitor alone or in combination reduced the expression of CXCL1, CXCL2, SDF1, and MCP1 in myeloma cells (**Figure 5C**). These results indicate that interaction with MSCs upregulates cytokine expression in myeloma cells *via* the integrin α6-mediated ERK1/2, Akt, STAT1, and STAT3 signaling pathways.

### Myeloma Cell Integrin α6 Regulates Osteoblast and Osteoclast Differentiation Through Binding to LAMA4 and EGFR on MSCs

**Figure 6:**
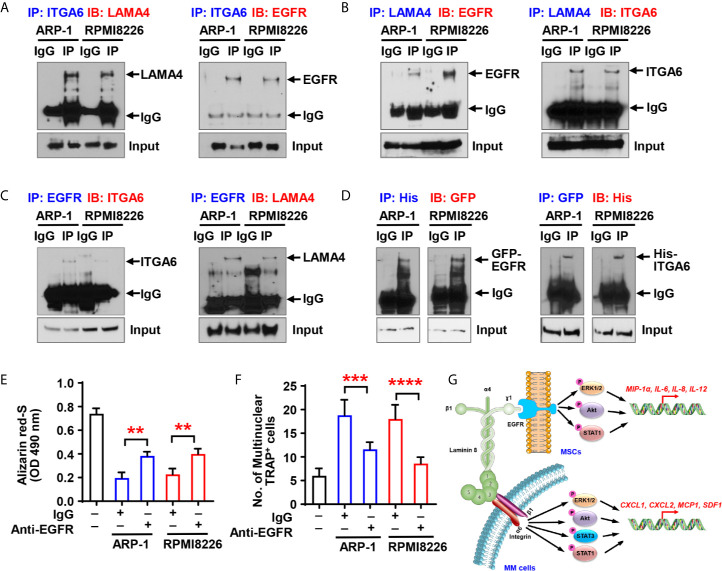
Myeloma cell integrin α6 regulates osteoblast differentiation *via* the ITGA6-LAMA4-EGFR complex. **(A–C)** Co-immunoprecipitation of ITGA6 **(A)**, LAMA4 **(B)** or EGFR **(C)** in myeloma cells ARP-1 or RPMI8226 cocultured with MSCs. **(D)** Myeloma cells were transfected with His-tagged integrin α6 and MSCs were transfected with GFP-tagged EGFR. Coimmunoprecipitation of His (left panel) or GFP (right panel) in myeloma cells and MSCs coculture system. **(E)** Shown is the summarized data of Alizarin red S staining of MSCs, which were pre-treated with IgG control or 2 µg/ml of anti-EGFR neutralize antibody and then co-cultured with or without ARP-1 or RPMI8226 cells for 14 days. **(F)** Shown is numbers of multinuclear (≥3) TRAP^+^ cells in preOCs that were pre-treated with IgG control or 2 µg/ml of anti-EGFR neutralize antibody and then co-cultured with or without ARP-1 or RPMI8226 cells for 7 days. Data shown as averages ± SD is a representative of three independent experiments. **P < 0.01. ***P < 0.001. ****P < 0.0001. *P* values were determined using one-way ANOVA. **(G)** Depiction of integrin α6-mediated signaling pathways in myeloma cells and MSCs.

Though we have demonstrated that there is a crosstalk between MSCs and integrin α6 from myeloma cells, a key piece of puzzle is notably missing - there is no known ligands of integrin α6 on MSCs, so we were wondering how myeloma cell integrin α6 communicates with MSCs. Previous studies have showed that integrin α6 can bind to the α4 subunit of laminin-8 (α4/β1/γ1), which is a major protein in the basal lamina and is highly expressed by MSCs (12). To examine the interaction between integrin α6 and laminin α4 in myeloma, we cocultured ARP-1 or RPMI8226 myeloma cells with MSCs and then immunoprecipitated the cell lysates with antibody against integrin α6. Western blotting showed the presence of laminin α4 in the immunoprecipitant (**Figure 6A**, left panel). Since the γ1 subunit of laminin-8 has been shown to contain an EGF-like sequence, which can bind to EGFR (13, 14), we pulled down the lysates of ARP-1 or RPMI8226 cells and MSCs using an anti-EGFR antibody and we detected either integrin α6 or laminin α4 proteins in immunoprecipitants (**Figure 6C**). As expected, we found the presence of EGFR in immunoprecipitants by an anti-integrin α6 antibody (**Figure 6A**, right panel) or an anti-laminin α4 antibody (**Figure 6B**, left panel). To further demonstrate the interaction between myeloma cells and MSCs through such complex, we transfected myeloma cells with His-tagged integrin α6 or transfected MSCs with GFP-tagged EGFR. Co-immunoprecipitation assays showed the presence of GFP-tagged EGFR or His-tagged integrin α6 proteins when we immunoprecipitated cell lysates using the antibody against His or GFP, respectively (**Figure 6D**). These results points to the formation of triple complex by myeloma cell integrin 6, MSC EGFR, and laminin 8. More specifically, laminin 8 serves as a connector between integrin 6 and EGFR, thereby enables the interaction between myeloma cells and MSCs.

To determine the functional role of MSC EGFR in myeloma-induced bone cell differentiation, we first cocultured MSCs and myeloma cells with the EGFR neutralizing antibody, washed out the antibody, and collected the CM. Addition of anti-EGFR antibody enhanced Alizarin red S staining in the cultures with the CM of MSCs cocultured with myeloma cells comparing to those with addition of IgG control, indicating that neutralizing EGFR reduced the inhibitory effect of myeloma cells on osteoblast differentiation (**Figure 6E**). We then cultured preOCs with the CM from coculturing MSCs with myeloma cells in the presence of anti-EGFR neutralizing antibody, and we observed less TRAP^+^ cells when EGFR were inhibited (**Figure 6F**), indicating that myeloma cell-mediated osteoclastogenesis can be eased by deactivation of EGFR. These results suggest the importance of the α6-LAMA4-EGFR complex in myeloma-induced osteoclastogenesis and suppression of osteoblastogenesis.

## Discussion

Our study reveals an important function of integrin α6 in the pathogenesis of myeloma-induced bone lesions − integrin α6 contributes to decreased osteoblastogenesis and increased osteoclastogenesis (**Figure 6G**). The formation of a trimer complex among myeloma cell integrin α6, laminin 8, and EGFR on MSCs activates the Akt, ERK, STAT1/3 signaling pathways, and therefore upregulates osteolytic cytokine expression in both myeloma cells and MSCs. These observations are important because they not only provide an insight into explaining a common pathogenic phenomenon in myeloma patients, but also suggest a therapeutic strategy for myeloma bone disease.

Integrin family members play an important role in normal bone remodeling. Integrins α5β1 and α_V_β3 have been shown to promote osteoblast differentiation *via* the FAK-ERK1/2 signaling and maintain osteoblast survival through activation of PI3K/Akt signaling (15). The integrin α_V_β3 is also essential for osteoclast attachment and osteoclast-mediated matrix degradation (16). In the tumor microenvironment, both integrins are shown to be involved in myeloma-induced bone lesions (3). The bone progenitor-expressed integrins α5β1 and α_V_β3 are the receptors of 2-deoxy-D-ribose (2DDR), of which we found to be highly secreted by myeloma cells. The secreted 2DDR binds to α5β1 and α_V_β3 on MSCs or preOCs, activates the PI3K/Akt signaling, leading to the DNA methyltransferase 3A-mediated hypermethylation during osteoclast and osteoblast differentiation (3). On the surface of myeloma cells, there are several integrins as well. For example, the integrin α4β1-expressed on myeloma cells has been shown to bind to vascular cell adhesion molecule-1 on MSCs (17). Such cell-cell contact stimulates the signaling to mediate myeloma-induced osteoclastogenic and bone-resorbing activity without influence induced by soluble cytokines. We have identified that integrin α6 is also highly expressed by myeloma cells (11). Distinct from other integrins, myeloma cell α6 forms a trimer complex with laminin 8 and EGFR on MSCs, and the complex enhances osteolytic cytokine production through α6-mediated signaling pathways in myeloma cells and through EGFR-mediated signaling in MSCs. Thus, integrin α6 is a new factor participating in the interaction between myeloma cells and MSCs, and such interaction-mediated osteoclast and osteoblast differentiation.

Several members of integrin family, including α1β1, α2β1, α3β1, α6β1, α7β1, and α6β4 heterodimers, serve as laminin receptors on a variety of cell types (18, 19). Laminins are large extracellular glycoproteins that involve in important biological processes, including tissue development, wound healing, and tumorigenesis. Laminins are heterotrimers containing α, β and γ chains. So far five α chains, three β chains and three γ chains have been identified, which leads to the formation of 16 laminin isoforms with distinct and tissue-specific functions (20–23). Previous research has revealed that laminin α chain interacts with integrins, leading to the phosphorylation of several critical tyrosine residues (23). In addition, it have shown a direct interaction between laminin γ chain and EGFR, and suggested that the interaction can activate the EGFR-mediated signaling (24). Our study, for the first time, demonstrates the integrin α6-laminin 8-EGFR complex that links myeloma cells and MSCs, and contributes to myeloma-induced osteolytic bone lesions. Since the bone marrow microenvironment is full of interactive players, such as osteoblasts, osteoclasts, and other stromal cells, the pathogenesis of myeloma associated-bone disease is very complicated. We believe that understanding of the crosstalk between integrin α6-mediated signaling and other pathways would be our logical next step. In this study, our results elucidate a new mechanism by which myeloma-MSC interaction upregulates the production of osteolytic cytokines through the integrin α6/laminin8/EGFR signaling and offer a potential strategy for targeted treatment of myeloma-associated bone disease.

## Data Availability Statement

The original contributions presented in the study are included in the article. Further inquiries can be directed to the corresponding authors.

## Ethics Statement

The animal study was reviewed and approved by Institutional Animal Care and Use Committee of UT MD Anderson Cancer Center and The Houston Methodist Hospital.

## Author Contributions 

HL and JY designed all experiments and wrote the manuscript. HL, ZW, JH, ZL, JG, and RL performed experiments and statistical analysis. PL provided patient samples. All authors contributed to the article and approved the submitted version.

## Funding

This research was supported by the National Institutes of Health/National Cancer Institute (R01 awards CA190863 and CA193362).

## Conflict of Interest

The authors declare that the research was conducted in the absence of any commercial or financial relationships that could be construed as a potential conflict of interest.
